# Maternal Age‐Related Gender Bias in Trisomy 21 and Trisomy 18

**DOI:** 10.1002/bdr2.2489

**Published:** 2025-06-02

**Authors:** Yun Pan, Changshui Chen, Haibo Li

**Affiliations:** ^1^ The Central Laboratory of Birth Defects Prevention and Control The Affiliated Women and Children's Hospital of Ningbo University Ningbo China; ^2^ Ningbo Key Laboratory for the Prevention and Treatment of Embryogenic Diseases The Affiliated Women and Children's Hospital of Ningbo University Ningbo China

**Keywords:** gender bias, maternal age, paternal diploid origin, trisomy 18, trisomy 21

## Abstract

**Background:**

The aim of this study was to investigate the underlying factors contributing to gender‐based disparities in the prevalence of trisomy 21 (Downs's syndrome) and trisomy 18 (Edwards's syndrome).

**Methods:**

Overall, 551 cases of trisomy 21 (T21) and 154 cases of trisomy 18 (T18) diagnosed through amniotic fluid karyotyping and chromosomal microarray analysis (CMA) between 2005 and 2023 at the Affiliated Women and Children's Hospital of Ningbo University. The study population consisted of fetuses at 19–23 gestational weeks across various maternal age groups. A control group comprising 662,453 newborns from the same institution between 2011 and 2018 was established for sex ratio comparison. Parental origin of diploids in T21 and T18 cases was determined using quantitative fluorescence‐polymerase chain reaction (QF‐PCR) analysis. Statistical significance of gender bias was evaluated using chi‐squared tests, with a threshold of *p* < 0.05 considered statistically significant.

**Results:**

The study revealed distinct sex ratio patterns across different maternal age groups. The control group exhibited a sex ratio of 1.06 (male:female), while the overall sex ratio for T21 cases was significantly elevated at 1.32. Notably, the highest sex ratio (1.84) was observed in T21 cases among women aged 20–25 years, with a progressive decline in sex ratio corresponding to increasing maternal age. The sex ratio of newborns born to women aged ≥ 35 years approximated that of the control. In contrast, T18 cases demonstrated an overall female predominance, with a sex ratio of 0.67, reaching its lowest value (0.56) in the 25–30 years maternal age group. Regarding the parent origin of diploids, maternal meiosis errors accounted for > 90% of cases in both T21 and T18. However, a higher prevalence of paternal origin was observed in younger women (≤ 35 years). Male fetuses of paternal diploid origin of T21 were 2.5 times more than female fetuses.

**Conclusion:**

In our sample of over 500,000 births, between 2005 and 2023 in Ningbo, China, fetuses with T21 were more likely to be males while fetuses with T18 were more likely to be females. However, this gender bias exhibited a significant age‐dependent pattern, being predominantly observed in women under 35 years of age. Specifically, in T21 cases of paternal origin among women ≤ 35 years, the frequency of nondisjunction involving Y‐chromosome‐bearing sperm was 2.5‐fold higher than that involving X‐chromosome‐bearing sperm.

## 1 Introduction

Trisomy 21 (T21) and trisomy 18 (T18) have been recognized as the most common autosomal trisomy syndromes since their discovery in 1959 (Lejeune et al. 1959) and 1960 (Edwards et al. 1960), respectively. Currently, most diagnoses are made prenatally based on maternal serological screening, noninvasive prenatal testing (NIPT) or amniocentesis, with pregnancy termination being the outcome in most cases. The live birth prevalence of T21 is 1/792 (de Graaf et al. 2015), while that of T18 ranges from 1/3600 to 1/10,000, with the best overall estimate being 1/6000 (Rasmussen et al. 2003; Root and Carey 1994). Studies have reported that the sex ratio (male/female) of T21 is 1.30 (Verma and Huq 1987), while that of T18 is 0.67 (Crider et al. 2008). More studies have focused on gender differences in the clinical phenotypes of Downs's syndrome (Iulita et al. 2023; Santoro et al. 2018).

As is well known, there is a slightly higher proportion of males in the human population (male:female = 106:100), regardless of race or region (D'Alfonso et al. 2023). However, data suggest that human male gametes carry an equal proportion of X and Y chromosomes during meiosis (Jongbloet 2005). Studies have shown that certain mutations can lead to gender bias (Kim et al. 2019). A recent study also identified gender bias in T21 and T18, but no clear conclusions could be drawn due to the unknown gender in more than 20% of cases (Zhang et al. 2023). Many retrospective studies have reported that T21 is more prevalent in males than in females, while T18 is more prevalent in females than in males (Crider et al. 2008; Verma and Huq 1987). However, the intrinsic causes of this gender disparity remain unknown.

## 2 Methods

### 2.1 Study Population and Data Collection

This study was conducted at the Affiliated Women and Children's Hospital of Ningbo University and approved by the hospital's ethics committee (EC2020‐014). Cases were collected from January 2005 to June 2023 through the hospital's prenatal diagnosis system. These cases met the following inclusion criteria: (1) maternal age ≥ 20 years; (2) fetal gender clearly documented in medical records; (3) complete medical records, including prenatal screening/diagnosis data, confirmed gestational age, and documented pregnancy outcomes. The materials primarily used for prenatal diagnosis included amniotic fluid and fetal villi. The testing methods mainly involved karyotyping and chromosomal microarray analysis (CMA), which were used to detect aneuploidy, copy number variations (CNVs), and uniparental disomy (UPD). The fetal sex ratios for trisomy 21 and trisomy 18 were calculated, and the sex ratio of newborns from our hospital between 2011 and 2018 served as the control. From 2011 to 2018, a total of 350,904 male babies and 311,549 female babies were born at the Affiliated Women and Children's Hospital of Ningbo University, resulting in a sex ratio of 1.06, which was used as the control group. Differences between the two groups were compared using chi‐squared tests, with a *p* value < 0.05 considered statistically significant. All analyzes were performed using SPSS 26.0 software.

### 2.2 Invasive Prenatal Diagnosis by Karyotyping and CMA

Samples from the chorionic villus and amniotic fluid of the fetuses were collected via ultrasound‐guided transabdominal chorionic villus sampling and amniocentesis. Cells were cultured for 10 days and then prepared for G‐banding karyotyping according to standard operating protocols. Genomic DNA was extracted from uncultured cells using the QIAamp DNA extraction kit (Qiagen, USA). The extracted DNA was then digested, amplified, purified, fragmented, labeled, hybridized, washed, and scanned using the Affymetrix CytoScan 750 k array chip. The offline data were analyzed using chAS 4.0 software, in combination with PubMed, ClinVar, Decipher, and other databases. The analysis was performed according to the guidelines of the American College of Medical Genetics and Genomics (ACMG) and the latest CNV pathogenicity guidelines from the Clinical Genome Resource Database (ClinGen).

### 2.3 Identify the Parent Origin of Diploids in Trisomy 21 and Trisomy 18 by QF‐PCR Amplification Combined With Capillary Electrophoresis

The amount of target sequence in the initial template is directly proportional to the amplification of short tandem repeats (STR) markers by QF‐PCR, which generates a fluorescent product. The AneuFilerTMV2 kit (Cubicise Medical, China), which includes thirty STR markers, was used to amplify selected microsatellites and the SRY region of the Y chromosome. This combination of markers allowed the detection of aneuploidies involving chromosomes 21, 18, 13, X, and Y. Chromosome 21 includes eight markers: D21S1437 (21q21.1), D21S2052 (21q21.1), D21S1446 (21q22.3), D21S1444 (21q22.13), D21S1432 (21q21.1), D21S1414 (21q21.1), D21S1411 (21q22.3), and Penta D (21q22.3). Chromosome 18 includes six markers: D18S862 (18q21.32), D18S878 (18q22.1), D18S391 (18q11.31), D18S867 (18q21.2), D18S865 (18q12.3), and D18S1371 (18q23). In a trisomic sample, three copies of a chromosome were detected by the presence of three peaks corresponding to chromosome‐specific markers, with equal fluorescent intensity and an area ratio of 1:1:1 (Trisomic Triallelic). In quantitative PCR, two chromosomes with the same repeat number produced two unbalanced fluorescent peaks with an area ratio of 2:1 (Trisomic Diallelic). The mother's peripheral blood sample was used as a control to assess maternal cell contamination based on the peak number (≤ 3) and to determine the parental origin of diploids using STR marker analysis.

## 3 Results

### 3.1 Gender Bias Was Related to Maternal Age

A total of 551 cases of T21 and 154 cases of T18 were diagnosed through karyotype analysis of amniotic fluid samples obtained from 37,190 pregnant women at 19–23 gestational weeks between 2005 and 2023. The sex ratios of these cases, stratified by maternal age, are summarized in Table 1. The sex ratio of T21 decreased with increasing maternal age. The highest sex ratio of 1.84 was observed in the 20–25 years age group, while the sex ratio approached normal levels when maternal age exceeded 35 years. The sex ratio of T21 in the 20–30 years maternal age group showed statistically significant differences compared to the sex ratio of the control (Table 1). For T18, the lowest sex ratio of 0.56 was observed in the 25–30 years age group, while the sex ratio tended to increase when maternal age exceeded 35 years. The sex ratio of T18 in the 25–30 years maternal age group showed statistically significant differences compared to the sex ratio of the control (Table 1).

**TABLE 1:** Sex ratios of T21 and T18 fetuses diagnosed with amniotic fluid karyotyping and chromosomal microarray analysis at different maternal ages from 19 to 23 gestational weeks (2005–2023).

Maternal age (years)	T21		Sex ratio	*p*	T18		Sex ratio	*p*
	Male	Female			Male	Female		
20–25	46	25	1.84	0.046	14	13	1.08	0.91
26–30	90	56	1.61	0.036	19	34	0.56	0.013
31–35	80	57	1.4	0.2	12	21	0.57	0.056
36–40	70	73	0.96	0.34	15	18	0.83	0.39
> 40	28	26	1.08	0.87	2	6	0.33	0.11
Together	314	237	1.32	0.059	62	92	0.67	0.002

### 3.2 Parent Origin of Diploid Was Critical to Gender Bias

To determine the causes of gender bias in T21 and T18, QF‐PCR based on STR was used to detect the parent origin of diploids of T21 and T18. More than 90% parent origin of diploids in T21 and T18 was maternal, with a higher proportion of paternal origins in young women (≤ 35 years). Male fetuses of paternal diploid origin of T21 were 2.5‐fold higher than female fetuses (Table 2).

**TABLE 2:** Parent origin of T21 and T18 diploids based on STR results.

Maternal age	Male			Female			Sex ratio*
	Parent origin of diploid						
	Maternal	Paternal	Unknown	Maternal	Paternal	Unknown	
T21							
≤ 35 years	23 (23%)	5 (5%)	6 (6%)	21 (21%)	2 (2%)	1 (1%)	1.41
> 35 years	22 (22%)	0	2 (2%)	16 (16%)	1 (1%)	1 (1%)	1.33
T18							
≤ 35 years	5 (25%)	0	0	6 (30%)	0	0	0.83
> 35 years	4 (20%)	0	0	5 (25%)	0	0	0.8

* The sex ratio is defined as the ratio of the total number of males to the total number of females.

## 4 Discussion

An adult human is estimated to consist of approximately 30 trillion mostly diploid cells, with millions of cell divisions occurring every minute (Bianconi et al. 2013). However, the spindle assembly checkpoint (SAC) has been regarded as the primary surveillance mechanism preventing chromosome segregation errors during mitosis and meiosis (Musacchio and Salmon 2007). The SAC is a critical cell cycle regulatory mechanism that delays chromosome segregation in response to unattached kinetochores. Given the vast number of cell divisions and the limited monitoring mechanisms, chromosome aneuploidy remains a possibility to some extent.

Studies have shown that in women of advanced age (> 35 years), the cohesin complex—a ring‐like structure essential for maintaining sister chromatid cohesion—undergoes age‐dependent degradation, along with crossover maturation inefficiency, leading to fewer meiotic crossovers on chromosomes (Wang et al. 2019). These age‐related effects contribute to a higher frequency of chromosome mis‐segregation in oocytes of advanced age women (> 35 years), leading to a significant increase in aneuploid eggs (Franasiak et al. 2014; Hassold and Hunt 2001; Nagaoka et al. 2012). We hypothesize that the mechanisms underlying aneuploid egg production differ between younger and older women. By calculating the sex ratios of T21 and T18 cases stratified by maternal age (Table 1), we observed that gender bias was present only in women ≤ 35 years old, with the sex ratio of T21 negatively correlating with maternal age (Table 1).

The male predominance in humans is thought to reflect an evolutionary adaptation to higher infant mortality rates among males (Cyrus Chu and Lee 2012). One possible cause for the gender bias in T21 is that a higher number of males may be required to ensure sufficient survivors to sustain the population. Although this concept lacks robust evidence, historical birth records consistently show a male preponderance, with a sex ratio (male/female) of 1.06 among 662,453 newborns at the Affiliated Women and Children's Hospital of Ningbo University. However, this theory fails to explain why T18 exhibits a female predominance.

We infer that the most likely cause of gender bias is paternal issues, as males are the sole contributors of the Y chromosome, which determines the sex of the offspring as male (XY) or female (XX). Furthermore, relevant studies have shown that air pollution may lead to an imbalance in the ratio of X‐chromosome‐bearing sperm to Y‐chromosome‐bearing sperm, deviating from the normal 1:1 ratio by affecting the quality of male semen (Radwan et al. 2018). It can be inferred that other environmental factors, whether physical or chemical, that affect semen quality may also potentially lead to an imbalance in the ratio of X‐ and Y‐chromosome‐bearing sperm.

Studies indicate that over 90% T21 babies had T21 because of maternal nondisjunction during meiosis (Hassold and Hunt 2001; Patterson 2009; Vranekovic et al. 2012). In other words, most T21 cases originate before zygote formation. We examined retrospective data on the parent origin of diploids in T21 and T18. The results revealed that paternal diploid in T21 was significantly more common in younger women (≤ 35 years) than in older women (> 35 years). Furthermore, among younger women, paternally derived nondisjunction involving Y‐chromosome‐bearing sperm was more frequent than that involving X‐chromosome‐bearing sperm (Table 2). This finding explains the male predominance in T21 cases among younger women. The intrinsic mechanism behind the higher proportion of Y‐chromosome‐bearing sperm in paternal nondisjunction remains unclear, but we speculate it may be related to the size of the chromosome, as both chromosome 21 and the Y chromosome belong to the G group. In contrast, we infer that paternal nondisjunction in T18 more frequently involves X‐chromosome‐bearing sperm, though insufficient cases limit definitive conclusions.

In summary, this study provides new insights into the gender bias observed in T21 and T18. Despite comprehensive data collection over 18 years, this single‐center, cross‐sectional study has limitations, including a potential lack of generalizability due to the hospital's geographic location. Nevertheless, our findings offer evidence to explain the causes of gender bias in T21, contributing to the broader understanding of paternal chromosome aneuploidy mechanisms.

## Ethics Statement

The current study was approved by the IRB of the Affiliated Women and Children's Hospital of Ningbo University (No. EC2020‐048). Signed informed consent was obtained from all participants in this study.

## Conflicts of Interest

The authors declare no conflicts of interest.

## Data Availability

All relevant data are within the manuscript.
